# Misdiagnosis of scrub typhus complicated by hemophagocytic syndrome

**DOI:** 10.1186/s12887-019-1475-x

**Published:** 2019-04-10

**Authors:** Miaomiao Lin, Airong Huang, Xiang Zheng, Lisha Ge, Shijun He

**Affiliations:** Second Affiliated Hospital of Wenzhou Medical University, Wen Zhou, China

**Keywords:** Scrub typhus, Hemophagocytic syndrome, Eschar, Misdiagnosis

## Abstract

**Background:**

This study sought to analyze the cases of clinical misdiagnosis of scrub typhus complicated by hemophagocytic syndrome.

**Methods:**

We retrospectively reviewed the medical records for diagnoses, clinical course, chest X-ray findings, laboratory data, and antibiotic therapy.

**Results:**

All nine patients were misdiagnosed at the outpatient department between 07/2009 and 07/2017. They were diagnosed with septicemia and hemophagocytic syndrome, sepsis and hemophagocytic syndrome, severe infection, hepatitis and hemophagocytic syndrome, or upper respiratory tract infection. Among the nine patients, hepatic function examination showed decreased albumin and elevated C-reactive protein levels in all patients; alanine aminotransferase was increased and platelets were decreased in eight patients. Weil-Felix reaction was positive in three of nine patients. Indirect immunofluorescence demonstrated positive IgM antibody and EB virus-IgM in all nine patients; Mycoplasma pneumoniae antibody was positive in seven patients. All nine patients underwent chest computed tomography; no abnormality was found in two patients. Patch shadow with increased density was found in seven patients, including four patients with right pleural effusion and two with bilateral pleural effusion. Bone marrow biopsy was performed in all nine patients and hemophagocytic cells were seen. The nine misdiagnosed cases were given multiple broad-spectrum antibiotics either successively or concomitantly before and after admission, but no effective antibiotics against *Orientis tsutsugamushi* were applied. After diagnosis was corrected to scrub typhus, five patients were switched to chloramphenicol and dexamethasone, two patients were given azithromycin and dexamethasone, and two patients were treated with chloramphenicol. Body temperature returned to normal within 2–3 days and the children were quickly relieved from their condition.

**Conclusion:**

Hemophagocytic syndrome may be the presenting clinical feature of scrub typhus and initially mask the disease. Initial misdiagnosis is common and includes septicemia and hemophagocytic syndrome. The eschar is a useful diagnostic clue and febrile patients without any localizing signs should be thoroughly examined for its presence.

## Background

Scrub typhus is a natural disease caused by *Orientis tsutsugamushi*. It Is transmitted by chigger bites, which are common in southeastern coastal areas and southwestern provinces and cities in Asia [1]. The severity of scrub typhus is highly variable among patients, ranging from mild symptoms to multiple organ failure and even death [2]. Scrub typhus is often misdiagnosed and there are 19 misdiagnoses for scrub typhus [3]. The rate of misdiagnosis is associated with the number of damaged organs, i.e., the more damaged organs, the higher the misdiagnosis rate is, which can be up to 71% [4]. Delayed diagnosis and neglect of scrub typhus might contribute to the misdiagnosis of scrub typhus [5]. When scrub typhus is complicated by hemophagocytic syndrome (HPS), clinical diagnosis and treatment are even more difficult, and the disease is prone to rapid deterioration and even death. Hence, this study aimed to summarize nine misdiagnosis cases of scrub typhus complicated by HPS.

## Methods

### Subjects

Between July 2009 and July 2017, case records of nine hospitalized children were scrutinized, including five males and four females, with age at onset of 11 months to 10 years. In terms of onset time, four cases occurred in July, four in August, and one in November. All children had a history of walking and playing on the grass before the onset.

### Diagnosis

Scrub typhus was diagnosed by published criteria [6], which are based on epidemiology, fever, specific eschar or ulcer, and serum indirect immunofluorescent assay (IFA) IgM ≥1:80. Meanwhile, the diagnostic criteria of HPS were those recommended by the Hematology group, Chinese Pediatric Society, Chinese Medical Association (2012) [7]. Once patients were diagnosed with scrub typhus, they underwent the routine tests for HPS such as blood routine, coagulation function, blood lipids, serum ferritin, T lymphocyte subsets, bone marrow puncture, etc., as well as other tests such as X-ray or chest CT, blood liver function, Weil-Felix reaction (reference titers for OX19, OX2, and OXK were all ≤ 1:160), indirect immunofluorescent assay, EB virus-IgM, *Mycoplasma pneumoniae* (by ELISA and PCR), etc.

### Clinical evaluation

The outpatient and admission diagnosis, revised diagnosis, location of skin eschar or ulcer, clinical and imaging findings, selection of antibiotics, bone marrow examination, imaging examinations, and laboratory examinations were evaluated.

## Results

### Clinical manifestations

**Fig. 1:**
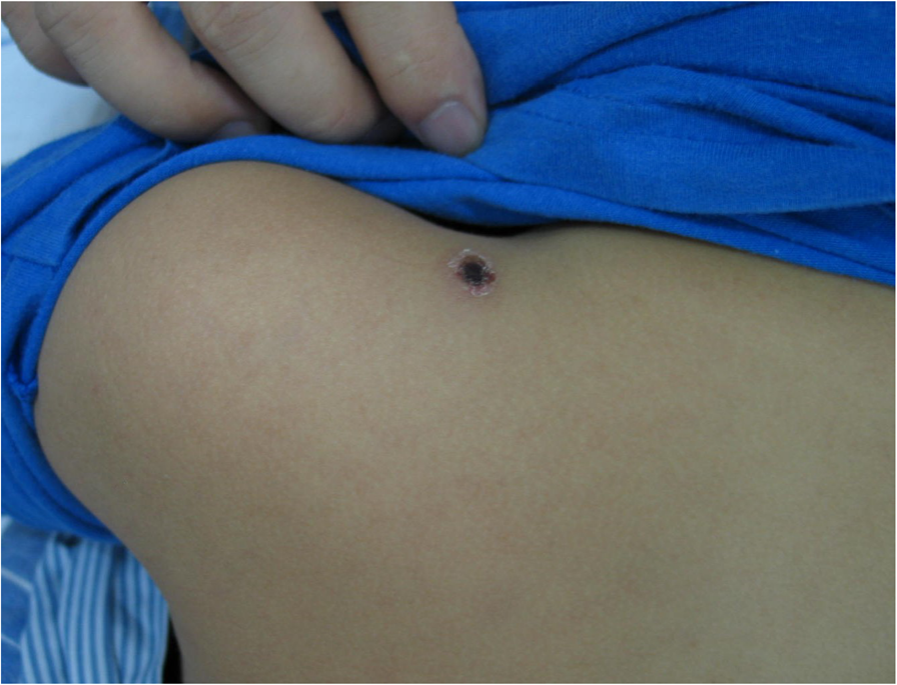
Eschar on the left shoulder

**Fig. 2:**
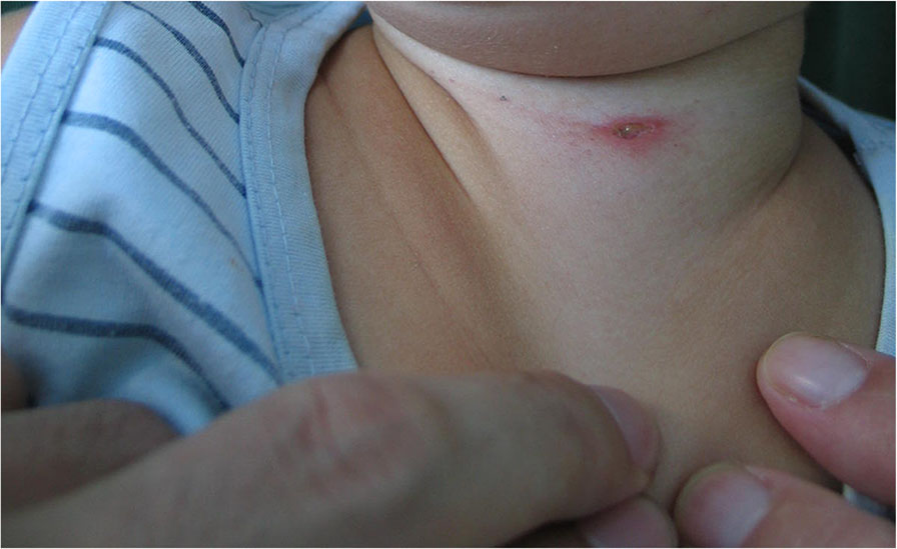
Ulcers in the fold of anterior cervical skin

All nine patients had fever. Two patients had erythema and four had mild cough. All patients were misdiagnosed at the outpatient department. Of these, three patients were diagnosed as septicemia and HPS; two were diagnosed as sepsis and HPS; three were diagnosed as severe infection, hepatitis and HPS; and one was diagnosed as upper respiratory tract infection. Among the nine cases, the eschar was subtle and was discovered after repeated and careful search. Then, the diagnoses were corrected as scrub typhus after 3 days of admission. One patient had an eschar in the left popliteal fossa and one on the left shoulder (Fig. 1). One patient had ulcers in the fold of the anterior cervical skin (Fig. 2), one on the scrotum, two in the inguinal area, two on the base of the thigh, and one in the fold of the auricle skin.

### Auxiliary examination

**Fig. 3:**
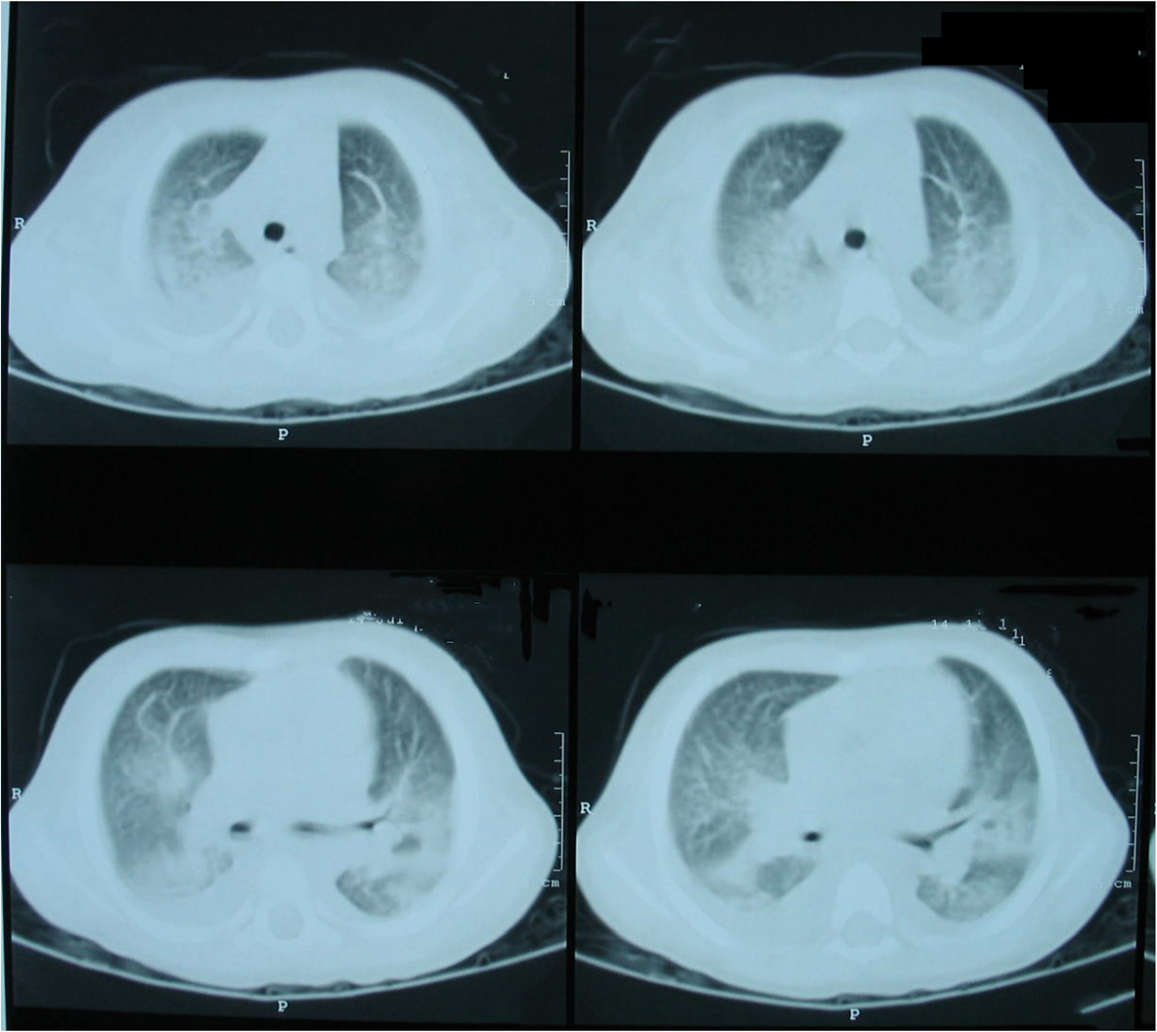
Two lung patch shadows with pleural effusion

**Fig. 4:**
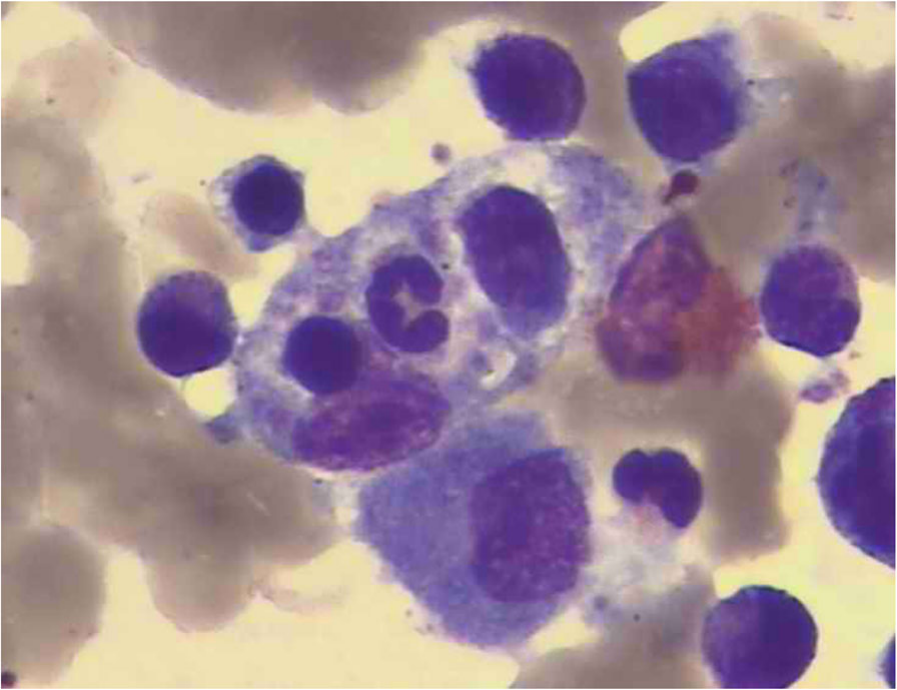
Bone marrow macrophage phagocytosis

In all nine patients, hepatic function examination revealed decreased albumin and elevated C-reactive protein levels; alanine aminotransferase was increased and platelets were decreased in eight patients (Table 1). Among the nine patients, Weil-Felix reaction was positive in three patients. Indirect immunofluorescent assay (reagent was a product of FOCUS, USA) demonstrated positive IgM antibody and EB virus-IgM in all nine patients, while *Mycoplasma pneumoniae* antibody was positive in seven patients. All nine patients underwent chest CT examination. No abnormality was found in two patients. Patch shadow with increased density was found in seven patients, including four with right pleural effusion and two with bilateral pleural effusion (Fig. 3). Bone marrow biopsy was performed in all nine patients, and hemophagocytic cells were seen in the bone marrow smears (Fig. 4).

**Table 1:** Related laboratory results of 9 children

Case	ALT (IU/L)	CRP (mg/L)	Hb (g/L)	Plt (× 10^9^/L)	ALB (g/L)	Blood fat (mmol/L)	Fib (g/L)	SF (ng/ml)	NK cell (%)
1	88	165	78	25	15.9	2.56	0.53	1500	5.55
2	132	72	80	56	29.6	1.77	2.12	568	5.96
3	96	160	81	90	24.6	2.14	2.66	468	12.7
4	810	105	109	34	25.5	2.94	1.35	1500	7.49
5	41	23	91	195	27.7	3.99	2.65	210	7.15
6	87	44	102	67	20.4	2.45	1.47	1500	6.54
7	90	78	87	55	18.3	2.09	1.96	408	7.35
8	66	39	54	89	23.2	3.15	2.24	587	5.98
9	25	95	48	44	26.0	3.07	2.16	768	7.90

### Treatment

The nine misdiagnosed cases were given multiple broad-spectrum antibiotics either successively or concomitantly before and after admission, but no effective antibiotics against *Orientis tsutsugamushi* were applied. Among them, one patient successively received ceftriaxone, cefoperazone sulbactam, and meropenem; one successively received ceftriaxone and penicillin; one received imipenem and cefoperazone sulbactam; three received ceftazidime; two received ceftriaxone; and one received erythromycin. All cases remained with persisting high fever despite treatments. After the diagnosis of scrub typhus, five patients were switched to chloramphenicol and dexamethasone; two were given azithromycin and dexamethasone; and two were treated with chloramphenicol. Body temperature returned to normal within 2–3 days and all children were quickly relieved from their condition.

## Discussion

Scrub typhus is caused by *Orientis tsutsugamushi* and is transmitted by chiggers. The disease is common in southeastern coastal areas and southwestern provinces and cities in Asia [8]. *Orientis tsutsugamushi* causes localized skin lesions at the site of the bite and enters the circulation directly or through the lymphatic system after approximately 10 to 14 days, and subsequently grows and proliferates in vascular endothelial cells and macrophages [9]. The basic pathological change of this disease is invasion of vascular endothelial cells, which causes extensive vasculitis and perivascular inflammation [10], and sometimes even involves multiple organs in severe cases. Typical clinical symptoms include fever, rash, eschar, and hepatosplenomegaly.

High numbers of involved visceral organs in patients with scrub typhus are associated with higher rates of misdiagnosis [4], and there are about 19 misdiagnosed diseases for scrub typhus [3]. There are many reasons for this. There is a low susceptibility that chiggers transmit scrub typhus, the characteristic eschar and ulcers are often hidden on the body, and there are complex clinical manifestations and atypical symptoms in cases of multiple organ damage, for which most of the pediatricians lack knowledge on scrub typhus [4] and prone to a rash diagnosis [11]. If the scrub typhus is complicated by HPS, the clinicians mostly diagnose the case as severe infection or HPS, and neglect primary scrub typhus. Therefore, there is a need for clinicians to carefully look for skin eschar, especially on hidden parts of the body. Nevertheless, it has to be noted that the presence of an eschar does not necessarily indicate the diagnosis of scrub typhus. The whole clinical portrait has to be taken into account. In addition, scrub typhus with HPS is most likely to be misdiagnosed. For the nine patients in the present study, eight cases were diagnosed as HPS at admission, not as scrub typhus. In 1992, Kobayashi first reported a case of a 47-year-old man who had prolonged hyperthermia. His condition remained progressive after penicillin treatment and he had decreased platelets and lymphocytes. Bone marrow biopsy revealed hematopoietic cells [12]. Subsequently, five cases of scrub typhus complicated with HPS were reported one after another [13, 14]. Other cases were reported in Taiwan and Guangdong in China, as well as in Japan [3, 14, 15]. Fifteen cases were reported in Taiwan, including two cases with HPS [16]. According to the literature, scrub typhus complicated by HPS is a rare occurrence. Nevertheless, our department has started routine bone marrow puncture in children with scrub typhus and bone marrow smears have revealed that the hemophagocytic phenomenon is not uncommon in scrub typhus.

To reduce the rate of misdiagnosis and achieve early and timely diagnosis of scrub typhus requires clinicians to fully understand the specific signs of the disease. The eschar is one of the most important clinical clue to diagnose scrub typhus [17]. The eschar is often difficult to find because it is painless and not itching, and it is more likely to be found when searching the vicinity of swollen lymph nodes [6]. The presence of an eschar was reported in 46–92% of the cases in South Korea, but in < 10% in Taiwan [17] and in 17% in India [18]. All nine patients included were initially misdiagnosed, and one of the reasons for misdiagnosis was that the eschar was not found. In all nine patients, the eschar was found in difficult-to-observe places after a careful examination by different pairs of eyes. It is therefore possible that the eschar was also missed in many patients in the previous studies. On the other hand, skin lesions located on humid sites such as the perineum and axilla [19] manifest as ulcers, which are prone to misdiagnosis and negligence [6]. Improving the vigilance of first-line clinical staffs is an effective way to improve the early diagnosis. Clinicians and nurses should receive training to perform repeated and detailed examinations and targeted laboratory tests for children with fever who are suspected of scrub typhus with multiple organ involvement. All nine patients in the present study were recorded as suspected scrub typhus shortly after admission. After careful and detailed examination of the entire body, even in hidden folds of the skin, the skin lesions were found and the diagnosis was corrected to scrub typhus. They then received effective treatment.

In terms of treatment, it is very important to use antibiotics during the early stage of the disease [20]. Studies showed that antibiotics in patients with scrub typhus complicated with acute respiratory distress syndrome (ARDS) were significantly delayed compared with the control group (patients with scrub typhus without ARDS) [onset (13.88 ± 4.19) vs. (9.25 ± 3.35) days] [20]. Another study suggested that the risk was increased by at least 20% if doxycycline was delayed for an average of 3 days, and patients often showed worsened symptoms or even died if they were untreated for 5 days after disease onset [17]. Antibacterial drugs are preferred for the treatment of scrub typhus with HPS, and body temperature may be restored within 3 days after oral administration of minocycline [13]. Doxycycline treatment can eliminate the phenomenon of hemophagocytosis [15]. Doxycycline, the antibiotic of choice for Scrub typhus, could not be given to the patients, due to its non-availability at that point of time. Further studies have found that in cases with scrub typhus and HPS, the levels of TNF-α, IFN-γ, and IL-10 were significantly reduced within 24 h after treatment with doxycycline, while the levels IL-1β, IFN-γ, and IL-10 mRNAs showed a decrease after 2–7 days of treatment; hence, the mechanism of rapid fever reduction after doxycycline treatment was consistent with the decreased of the inflammatory factors [21]. One case was reported to have been treated with doxycycline+glucocorticoids for scrub typhus with HPS [15]. In the present study, two out of nine patients were treated with chloramphenicol alone and five patients were treated with dexamethasone and chloramphenicol. All patients showed rapidly resolving fever.

The present study has limitations. The sample size was small and from a single center. In addition, because of the retrospective nature of the study, some test results were unavailable. Additional studies are necessary to examine the diagnosis of scrub typhus.

## Conclusion

Hemophagocytic syndrome may be the presenting clinical feature of scrub typhus and initially mask the disease. Initial misdiagnosis was common and included septicemia and hemophagocytic syndrome. The eschar is a useful diagnostic clue and febrile patients without any localizing signs should be thoroughly examined for its presence.

## Abbreviations

HPS: Hemophagocytic syndrome
IFA: Immunofluorescent assay
